# Microbial larvicides for mosquito control: Impact of long lasting formulations of *Bacillus thuringiensis* var. *israelensis* and *Bacillus sphaericus* on non‐target organisms in western Kenya highlands

**DOI:** 10.1002/ece3.4250

**Published:** 2018-07-06

**Authors:** Yahya A. Derua, Samuel C. Kahindi, Franklin W. Mosha, Eliningaya J. Kweka, Harrysone E. Atieli, Xiaoming Wang, Guofa Zhou, Ming‐Chieh Lee, Andrew K. Githeko, Guiyun Yan

**Affiliations:** ^1^ Kilimanjaro Christian Medical University College Tumaini University Makumira Moshi Tanzania; ^2^ National Institute for Medical Research Amani Research Centre Tanga Tanzania; ^3^ Department of Zoology School of Pure and Applied Sciences Pwani University Kilifi Kenya; ^4^ Division of Livestock and Human Diseases Vector Control Tropical Pesticides Research Institute Arusha Tanzania; ^5^ Department of Medical Parasitology and Entomology Catholic University of Health and Allied Sciences Mwanza Tanzania; ^6^ School of Public Health Maseno University Kisumu Kenya; ^7^ Program in Public Health College of Health Sciences University of California Irvine California; ^8^ Climate and Human Health Research Unit Centre for Global Health Research Kenya Medical Research Institute Kisumu Kenya

**Keywords:** aquatic vertebrates and invertebrates, *Bacillus sphaericus*, *Bacillus thuringiensis* var. *israelensis*, larviciding

## Abstract

The microbial larvicides *Bacillus thuringiensis* var. *israelensis* and *Bacillus sphaericus* have been used extensively for mosquito control and have been found to be effective and safe to non‐target organisms cohabiting with mosquito larvae. Recently developed long lasting microbial larvicides (LLML), although evading the previous challenge of short duration of activity, increase the risk of persistence of toxins in the treated larval habitats. This study monitored the impact of LLML FourStar^®^ and LL3 on non‐target organisms cohabiting with mosquito larvae in an operational study to control malaria vectors in western Kenya highlands. A total of 300 larval habitats were selected in three highland villages. The habitats were first monitored for 5 weeks to collect baseline data on non‐target organisms cohabiting with mosquito larvae and then randomized into two treatment arms (respective FourStar^®^ and LL3) and one control arm. Non‐target organisms were sampled weekly for 5 months after treatment to assess the impact of LLML intervention. Before treatment, the mean density of all non‐target organisms combined in the control, LL3 and FourStar^®^ treated habitats was 1.42, 1.39 and 1.49 individuals per habitat per sampling occasion, respectively. Following treatment, this density remained fairly unchanged for 21 weeks at which time it was 1.82, 2.11, and 2.05 for the respective control, LL3 and FourStar^®^ treated habitats. Statistical analysis revealed that LL3 and FourStar^®^ did not significantly alter abundance, richness or diversity of the 11 taxa studied, when comparing the intervention and control larval habitats. However, both FourStar^®^ and LL3 significantly reduced the density of malaria vectors. In conclusion, one round of label rate application of FourStar^®^ or LL3 in natural larval habitats did not alter richness, abundance or diversity of the monitored aquatic non‐target organisms cohabiting with mosquito larvae to an ecologically significant level.

## 1 INTRODUCTION

Mosquitoes breed in a variety of aquatic habitats and have a global distribution. Despite of their important role in the ecosystem, some species are also important disease vectors that spread malaria and other parasites as well as arboviruses (Fang, 2010). Malaria has thus remained an important human mosquito‐borne disease, and in 2016 it was estimated that 216 million cases of human malaria occurred worldwide, resulting into 445,000 deaths (WHO, 2017). At present, malaria control relies heavily on the use of long lasting insecticide treated nets (LLINs) and/or indoor residual spraying with insecticide (IRS) to control the vectors (WHO, 2017). However, widespread insecticide‐based interventions have resulted in evolution of insecticide resistance to all classes of insecticides used for malaria vectors (Butler, 2011; WHO, 2017). As an adaptation to the insecticidal pressure, malaria vectors have moreover been observed to change their biting and resting behavior (Moiroux et al., 2012; Sougoufara, Doucouré, Sembéne, Harry, & Sokhna, 2017; Sougoufara et al., 2014). Malaria vector shift and replacement have also been reported following IRS intervention (Gillies & Furlong, 1964; Gillies & Smith, 1960). For the continued delivery of effective insecticide‐based interventions for malaria control, there is a need to develop more ecologically friendly alternatives with a potential to evade adaptation mechanisms by the vectors.

Mosquito larvae control has a proven record of lowering malaria transmission or even eradication of malaria mosquitoes (Killeen, Fillinger, Kiche, Gouagna, & Knols, 2002). It has been observed that unlike adult mosquitoes, larvae do not change their behavior to avoid control interventions targeted at larval habitats (Killeen, Fillinger, & Knols, 2002). Moreover, larvae control strategy also serves to extend the useful life of insecticides by reducing selection pressure for resistance development and the strategy is equally effective in controlling both indoor and outdoor biting mosquitoes. An integrated approach of larval control with adult mosquito control interventions like LLINs and IRS has been considered to be a highly effective method for control of malaria (Walker & Lynch, 2007).

Larviciding with chemical agents has been a historically important component of malaria vector control (Killeen, Fillinger, Kiche, et al., 2002). However, due to significant adverse effects to other non‐target species, chemical larvicides have received gradually less attention in the past decades. Instead, preference has shifted to the use of microbial larvicides *Bacillus thuringiensis* var. *israelensis* (Bti) and *Bacillus sphaericus* (Bs) which selectively kill mosquito larvae with negligible effect to the non‐target organisms (Walker & Lynch, 2007). Susceptible mosquito larvae have alkaline gut conditions, enzymes and specific receptors for processing and binding of the Bti or Bs toxins (Baumann, Clark, Baumann, & Broadwell, 1991; Bravo, Gill, & Soberón, 2007; Dadd, 1975; Nicolas, Lecroisey, & Charles, 1990; Soberón, Fernández, Pérez, Gill, & Bravo, 2007). Thus, the toxins responsible for the pathogenic effect in mosquito larvae have no effect to vertebrates and some invertebrates, and hence they are suitable for application even in peri‐domestic mosquito breeding habitats (Lacey, 2007; Lacey & Merritt, 2003; Saik, Lacey, & Lacey, 1990). However, the conventional Bti and Bs have low residual activity and require repeated applications, which increase the cost of interventions (Fillinger, Knols, & Becker, 2003; Majambere, Lindsay, Green, Kandeh, & Fillinger, 2007; Majambere et al., 2010). In the recent past, long lasting microbial larvicide formulations that combine both Bti and Bs with potential for sustained release of active ingredients for up to 6 months have become available (Afrane et al., 2016; Zhou et al., 2016). The longer duration of activity may result in longer persistence of the toxin crystals in the environment and ultimately this may have direct or indirect adverse effects on non‐target organisms cohabiting with the mosquito larvae (Dupont & Boisvert, 1986).

A variety of non‐target organisms has been found to coexist with the mosquito fauna in aquatic habitats (Bukhari, Takken, Githeko, & Koenraadt, 2011; Fillinger, Sombroek, et al., 2009; Kweka, Zhou, Gilbreath, et al., 2011; Service, 1977) and to play a critical role in regulating the aquatic stages of mosquitoes through predation and competition. Diverse orders of aquatic vertebrates and invertebrates prey on mosquito larvae (Kweka, Zhou, Gilbreath, et al., 2011; Ohba et al., 2010). In addition to direct predation, the predators cause considerable indirect impacts on the population dynamics of the prey species (Åbjörnsson, Brönmark, & Hansson, 2002; Lima, 1998). Studies have shown that increased anti‐predator behavior such as avoiding colonizing habitats with predators translates into increase in duration of gonotrophic period of the prey and hence in a reduction in reproductive output (Åbjörnsson et al., 2002; Bond, Arredondo‐Jiménez, Rodríguez, Quiroz‐Martínez, & Williams, 2005; Lima, 1998; Petranka & Fakhoury, 1991). Anti‐predator behavior has also been linked with reduced energy intake and long‐term survival of the prey. On the other hand, the presence of co‐occurring species that compete for resources has been found to lower reproductive rates and survival of mosquito larvae (Kiflawi, Blaustein, & Mangel, 2003; Spencer, Blaustein, & Cohen, 2002).

Previous studies have suggested that microbial larvicides based on Bti and Bs are harmless to nearly all non‐target organisms when applied at recommended dosages (Lacey, 2007; Lacey & Merritt, 2003). However, the observation that Bti toxic crystals may persist in the environment has raised some concern that intensive applications could lead to accumulation of toxins with adverse effect on non‐target organisms (Boisvert & Boisvert, 1999; Dupont & Boisvert, 1986; Paris et al., 2011; Tilquin et al., 2008). An extensive review of the effect Bti on target and non‐target organisms has listed a number of studies indicating some negative effects on non‐target organisms (Boisvert & Boisvert, 2000). Other studies have suggested that by removing the target organisms, an important segment of the food web is removed, thereby possibly reducing ecosystem diversity and potentially altering the overall community structure (Hershey, Lima, Niemi, & Regal, 1998; Merritt, Wipfli, & Wotton, 1991). Monitoring the impact of Bti and Bs on non‐target organisms should, therefore, be an important requirement for mosquito control interventions using microbial larvicides. On this background, this study monitored the safety of long lasting microbial larvicides (LLML) based on Bti and Bs on non‐target organisms when used at a recommended dosage for 5 months of their duration of activity.

## 2 METHODS

### 2.1 Study area

**Figure 1:**
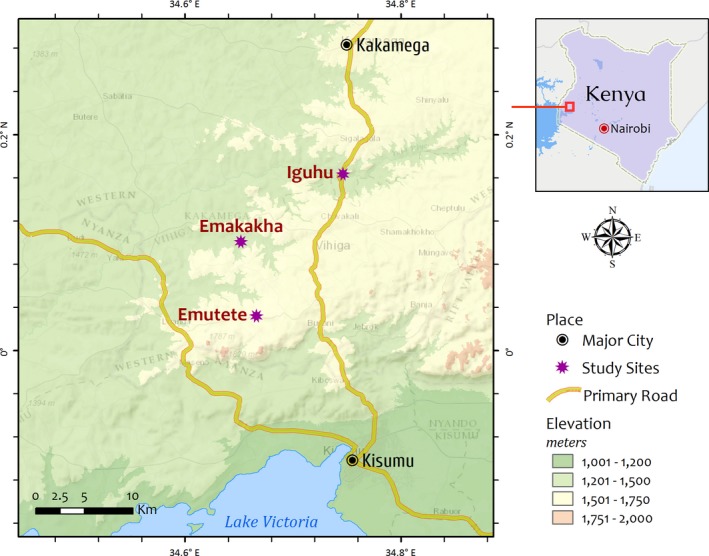
Location of study villages in Western Kenya Highlands

The study was conducted in three villages of western Kenya highlands. These were Iguhu (0.16176N, 34.76160E) in Kakamega County and two neighboring villages of Emutete (0.02627N, 34.61663) and Emakakha (0.10877N, 34.65331E) in Vihiga County (Figure 1). These villages have fairly the same topography and weather conditions and inhabitants practice subsistence farming and livestock keeping. The average annual rainfall is about 1,950 mm, with peak generally occurring between March and June followed by a short rainy season in October and November. The study area has been categorized as moderately endemic for malaria and epidemics are not uncommon (Hay et al., 2002). Detailed information on topography, weather conditions, human settlements and agricultural activities undertaken in the study villages have been described elsewhere (Minakawa, Munga, et al., 2005; Minakawa, Sonye, & Yan, 2005; Ndenga, Simbauni, Mbugi, & Githeko, 2012; Ndenga, Simbauni, Mbugi, Githeko, & Fillinger, 2011; Zhou et al., 2016).

### 2.2 Test materials

Newly developed LLML formulations FourStar^®^ briquets (Central Life Sciences, Sag Harbor, NY, USA) and LL3 (University of California, Irvine, CA, USA) were evaluated for their safety to non‐target organisms cohabiting with mosquito larvae. FourStar^®^ LLML formulation contains 1% *Bacillus thuringiensis* subspecies *israelensis* (Bti) Strain BMP 144 (potency 70 ITU [International Toxic Units]/mg), 6% *Bacillus sphaericus* (Bs) 2362, Serotype H5a5b, Strain AML614 (potency 60 ITU/mg) and 93% of other inert ingredients used to make briquets. The LL3 briquets has essentially the same contents and potency like FourStar^®^, the difference being that the inert ingredients used to make the former allows it to float (density approximately 0.99 g/cm^3^) once applied to the water body while the later sinks. According to the manufacturer's, once applied to the larval habitat, FourStar^®^ and LL3 briquets sustain release of effective levels of Bti and Bs to the water as the briquettes dissolve to effect mosquito larvae control for up to 180 days.

### 2.3 Experiments

**Figure 2:**
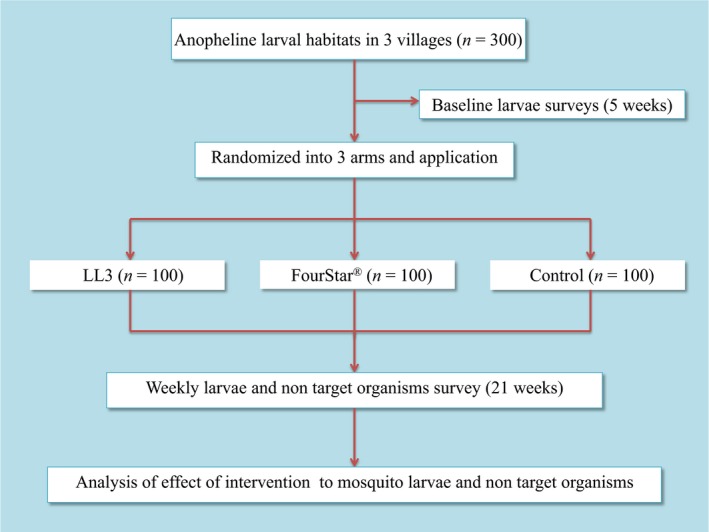
Study design

At the beginning of the study, 300 anopheline larval habitats were identified in the three selected villages and characterized based on previous classification (Kweka, Munga, Himeidan, Githeko, & Yan, 2015; Kweka, Zhou, Lee, et al., 2011). In brief, larvae habitats were classified by habitat type (drainage ditches, abandoned gold mines, ponds, fish ponds, roadside canals, rock pools, and swamps) and then identified using unique numbers. Baseline information on non‐target organisms was collected weekly from December 2015 to January 2016. The breeding habitats were then randomized (random number generator, Microsoft Excel 2007) into two intervention arms (treated with LL3 and FourStar^®^, respectively) and one control arm (nontreated habitats). From January 2016, FourStar^®^ and LL3 briquets were broadcasted by hand in the intervention habitats according to manufacturer's recommended dosage of one briquet for up to 100 square feet of surface area of the breeding habitat regardless of water depth. Following application, the impact of the treatment on non‐target organisms was monitored after 24 hr, 3 days, and weekly for up to 5 months, which roughly corresponds to the duration of activity of FourStar^®^ and LL3 briquets used (Figure 2). Non‐target organisms were surveyed using aquatic insect nets (Bioquip Products Inc, 2321 E. Gladwick ST. Rancho Dominguez CA) by gently dragging the net along the water surface at the margin of larval habitats as previous described (Ndenga et al., 2012). A 350 ml mosquito dipper was used for surveys in larval habitats with relatively high vegetation cover as aquatic insect nets proved to be impractical in those habitats. Particular attention was devoted to non‐target organisms with a potential role as predators or competitors of mosquito larvae. The collected non‐target organisms were classified to order and common names as described in the past (Bukhari et al., 2011; Fillinger, Sombroek, et al., 2009).

### 2.4 Data analysis

Data were entered in Excel and later transferred to R 3.3 for windows. Gini‐Simpson diversity index at each observation occasion was calculated for each site, and the average was calculated for each order of organisms and by treatment type (control, treatment with FourStar^®^ or LL3 LLML). The differences in abundance of each organism observed and in diversity were compared using generalized estimating equations (GEE) based on a Poisson distribution assumption in which baseline (binary) and observation time (in week) were treated as covariates. The values of the covariates were constant for the repeated elementary observations at each habitat. The correlation was tested against four assumptions, that is, independent, exchangeable, lag 1 autoregression (AR1) and unstructured. The models were first run using the interventions against control to evaluate the impact of interventions on the abundance of different organisms, then interventions against each other to determine the difference between the two LLML formulations. The same model and same process were further performed on the abundance of each organism and on the diversity at different habitat types to determine whether the impact was habitat‐dependent. Taxa richness in different larval habitats surveyed and among the three experimental arms was compared by Chi‐square test. *p*‐value < 0.05 was considered statistically significant.

### 2.5 Ethics

The study received ethical approval from the Scientific and Ethical Unit of the Kenya Medical Research Institute (Ref: KEMRI/RES/7/3/1). Before fieldwork, meetings were held with the respective County leaders to inform them about the study and to seek their cooperation. Oral informed consent was sought and obtained from land/farm owners before start of larval habitat surveys and application of LLML.

## 3 RESULTS

The impact of FourStar^®^ and LL3 LLML on non‐target organisms was monitored in 300 mosquito larval habitats, randomly assigned equally to either the two treatments or control. Environment conditions of the larval habitats were fairly similar between intervention and control arms. The habitats were followed once weekly for 26 (5 pre‐ and 21 post‐treatment) weeks. In each weekly round of monitoring, a mean of 289 (range 257–300) larval habitat were surveyed as some larval habitats dried or were destroyed by human activities and/or flooded by rains. The majority of the surveyed larval habitats were drainage ditches (38.0%), abandoned gold mines (30.1%) and ponds (20.8%). Other types of larval habitats surveyed were swamps, roadside canals, rock pools and fish ponds, contributing 3.5%, 1%, 1%, and 5.6% of all habitat types, respectively.

A total of 128,246 non‐target organisms belonging to 11 taxa were collected and identified. The taxa comprised of ephemeroptera (Mayfly nymphs), odonata (damselfly nymphs, dragon fly nymphs), hemiptera (water scorpion, water striders, water boatmen, backswimmers and water measurers), coleoptera (water beetles larvae and adults), diptera (biting flies and horse flies), arachnida (water mites, water spiders), molluscs (snails), annelida (leech, earthworm, flatworm), fish (tilapia, gambusia), amphibia (tadpoles, frogs), and decapoda (crabs). The collected non‐target organisms were dominated by five taxa of organisms namely: amphibians, hemipterans, coleopterans, odonata, and annelids, with overall mean densities per habitat per sampling occasion being 6.76, 3.95, 1.91, 1.77, and 1.17, respectively (Table 1). The non‐target organisms were more abundant in the abandoned gold mines and fish ponds, with overall mean densities of combined organisms per habitat type per sampling occasion being 2.51 and 1.80, respectively.

**Table 1:** Total abundance and mean ± *SE* of non‐target organisms (mean per habitat per sampling round) in control and LLML treated mosquito larval habitats in western Kenya highlands

Taxa	Common names	Total	Control	LL3	FourStar^®^
Fish	Tilapia, Gambusia	5,351	0.60 ± 0.05	1.00 ± 0.07	0.70 ± 0.05
Amphibians	Frogs, Tadpoles	47,116	6.52 ± 0.36	7.36 ± 0.41	6.41 ± 0.38
Molluscs	Snails	2,277	0.35 ± 0.02	0.30 ± 0.02	0.33 ± 0.02
Decapoda	Crabs	109	0.01 ± 0.00	0.02 ± 0.00	0.02 ± 0.00
Annelida	Leech, earthworms, flatworms	8,121	1.11 ± 0.04	1.21 ± 0.05	1.17 ± 0.04
Odonata	Damselfly nymphs, dragonfly nymphs	12,299	1.64 ± 0.05	1.90 ± 0.05	1.75 ± 0.05
Ephemeroptera	Mayfly nymphs	6,376	0.91 ± 0.04	0.91 ± 0.05	0.93 ± 0.04
Hemiptera	Water striders, water scorpions, water boatmen, water measurers, backswimmers	27,529	3.98 ± 0.10	3.98 ± 0.11	3.89 ± 0.10
Coleoptera	Water beetles	13,281	2.04 ± 0.16	1.82 ± 0.12	1.86 ± 0.13
Arachnida	Water mites, water spiders	5,114	0.74 ± 0.03	0.75 ± 0.03	0.71 ± 0.03
Diptera	Biting flies, horse flies	673	0.08 ± 0.02	0.11 ± 0.03	0.10 ± 0.03

**Figure 3:**
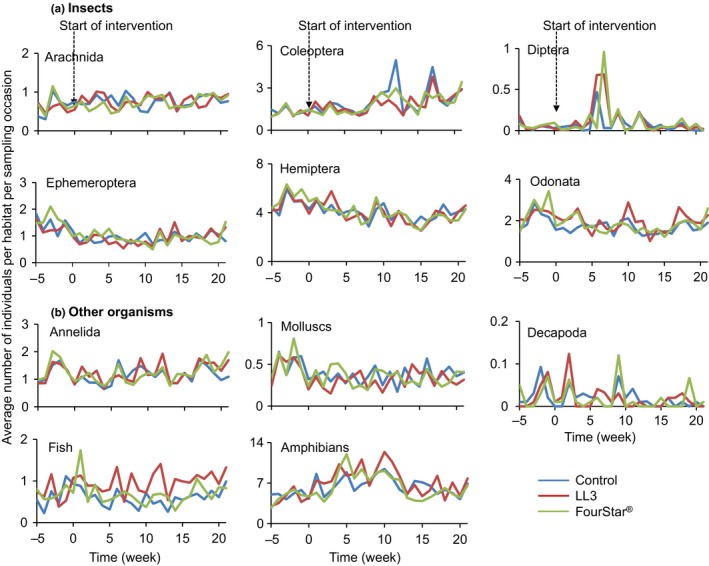
Abundance of individual taxa of non‐target organisms in treated and control mosquito larval habitats (a: insects; b: other organisms)

**Figure 4:**
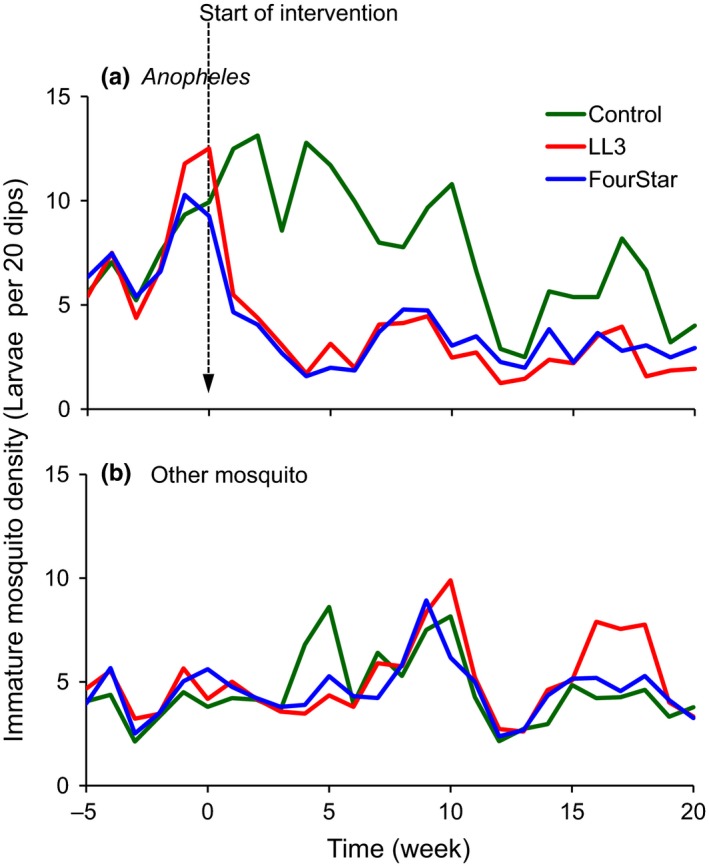
Impact of LLML on immature stages of mosquito. (a) Anopheles mosquitoes (*Anopheles gambiae* complex and *An. funestus* group) and (b) Other mosquito species combined

Prior to application of LLML (day 0), the mean density of all non‐target organisms combined in the control, LL3 and FourStar^®^ selected habitats was 1.42, 1.39, and 1.49, respectively. One week post‐treatment, the overall mean density of all non‐target organisms combined increased slightly (but not statistically significant) to 1.89, 1.91, and 1.71 individuals per habitat per sampling occasion for the respective control, LL3 and FourStar^®^ treated habitats. The trend of insignificant decrease or increase in density of combined non‐target organisms was maintained for 21 weeks in which time the mean density was 1.82, 2.11, and 2.05 for control, LL3 and FourStar^®^ treated habitats, respectively. Analysis for any change in the abundance of non‐target organisms over time revealed that the mean density of non‐target organisms surveyed was not significantly different (GEE, *p* > 0.1; Table 2; Figure 3) in intervention and control larval habitats after application of either FourStar^®^ or LL3 LLML. On the other hand, the mean density of individual taxa of non‐target organisms was not significantly different in the three arms of the study (GEE, *p* > 0.1; Table 1). However, FourStar^®^ and LL3 LLML significantly reduced the density of anopheline mosquitoes (GEE, *p* < 0.001; Figure 4; Table 2). The two interventions had no significant impact on non‐anopheline mosquitoes (GEE, *p* > 0.1; Figure 4). Comparison of the activity of the two LLML indicated that FourStar^®^ and LL3 were equally effective to the target and safe to the non‐target organisms (Table 2; Figures 3 and 4).

**Table 2:** Comparison of density of surveyed organisms between control and interventions and between the two interventions: *p*‐value calculated based on GEE models with Poisson distribution, exchangeable correlation and adjusted with baseline

Organisms	Order or family	Interventions vs. control		LL3 vs. FourStar^®^
		LL3	FourStar^®^	
Insects	Arachnida	n.s.^a^	n.s.	n.s.
	Coleoptera	n.s.	n.s.	n.s.
	Diptera b	n.s.	n.s.	n.s.
	Ephemeroptera	n.s.	n.s.	n.s.
	Heteroptera	n.s.	n.s.	n.s.
	Odonata	n.s.	n.s.	n.s.
	Culicidae^c^	<0.001	<0.001	n.s.
Other organisms	Annelida	n.s.	n.s.	n.s.
	Molluscs	n.s.	n.s.	n.s.
	Decapoda	n.s.	n.s.	n.s.
	Amphibians	n.s.	n.s.	n.s.
	Fish	n.s.	n.s.	n.s.

*Note*

^a^Not significant (*p* > 0.1). ^b^Excluding Culicidae. ^c^ *Anopheles* mosquitoes only.

**Figure 5:**
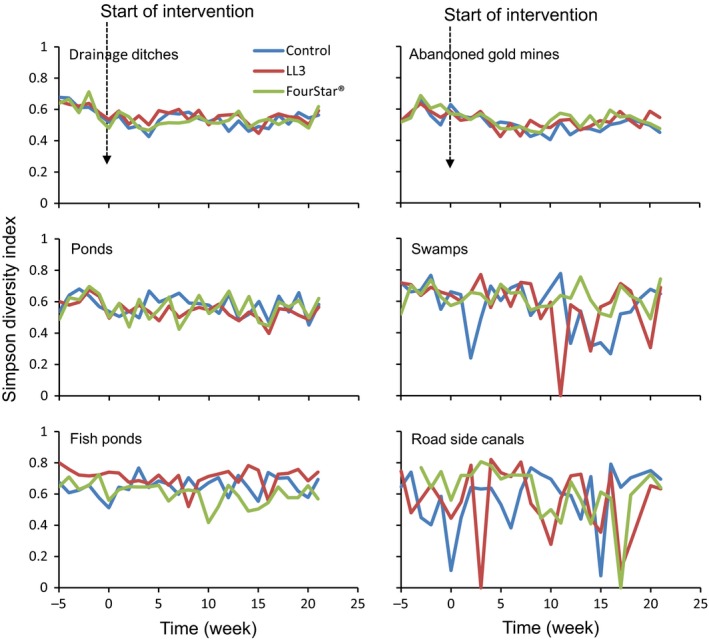
Diversity of non‐target organisms surveyed in different mosquito larval habitat types

The community structure of the 11 taxa of non‐target organisms was monitored in the different larval habitat for 21 weeks after LLML application. Prior to application of LL3 LLML, Simpson diversity index value for combined taxa of non‐target organisms in larval habitats was 0.54, 0.82, 0.74, 0.64 and 0.77 for drainage ditches, abandoned gold mines, ponds, swamps, and fish ponds, respectively. Twenty‐one weeks post application of LL3 LLML, the corresponding Simpson diversity index value for the respective larval habitats were 0.59, 0.77, 0.71, 0.71 and 0.79. For the FourStar^®^ LLML, Simpson diversity index value for non‐target organisms combined in drainage ditches, goldmines, ponds, swamps and fish ponds prior to the treatment was 0.48, 0.81, 0.76, 0.69, and 0.78, respectively. Twenty‐one weeks after application of the FourStar^®^ LLML, Simpson diversity index value for non‐target organisms for the respective larval habitats were 0.62, 0.76, 0.85, 0.79, and 0.80. Analysis by habitat types revealed that diversity of taxa of non‐target organisms as expressed by Simpson diversity index was not significantly different in the treated and control larval habitats (GEE, *p* > 0.1; Figure 5; Table 3). Likewise, taxa richness before and after application of FourStar^®^ or LL3 LLML, and between treated and control larval habitats were not significantly different (Table 3).

**Table 3:** Taxa richness and Simpson diversity index of the non‐target organisms in surveyed larval habitats prior and after application of LLML

Habitat types	Survey date (weeks)	Taxa richness^a^			Simpson Diversity Index		
		Control	LL3	FourStar^®^	Control	LL3	FourStar^®^
Drainage ditches	0^b^	9	10	11	0.52	0.54	0.48
	7	9	10	9	0.57	0.60	0.51
	14	9	9	9	0.46	0.50	0.48
	21	9	9	9	0.56	0.59	0.62
Abandoned gold mines	0^b^	10	10	10	0.63	0.58	0.57
	7	9	11	10	0.49	0.43	0.49
	14	10	9	10	0.47	0.49	0.56
	21	9	10	10	0.45	0.55	0.48
Ponds	0^b^	9	9	9	0.54	0.49	0.50
	7	10	8	9	0.65	0.50	0.42
	14	9	8	9	0.52	0.53	0.63
	21	8	8	9	0.58	0.57	0.62
Swamps	0^b^	6	3	6	0.66	0.64	0.57
	7	7	5	9	0.71	0.72	0.66
	14	4	4	7	0.31	0.28	0.61
	21	7	5	8	0.65	0.69	0.74
Roadside canals	0^b^	2	5	4	0.59	0.56	0.74
	7	3	4	2	0.38	0.71	0.72
	14	4	5	3	0.44	0.73	0.56
	21	4	5	0	0.75	0.65	0.73
Fish ponds	0^b^	8	7	10	0.51	0.74	0.56
	7	9	7	9	0.60	0.72	0.61
	14	9	9	9	0.64	0.78	0.49
	21	9	8	8	0.69	0.74	0.57

*Note*

^a^Analysis of taxa richness recorded lack of taxa variation in the three experimental arms (Chi‐square test, *p*‐value ranging from 0.96 to 1.0). ^b^Before LLML application.

## 4 DISCUSSION

Vector control with microbial larvicides is a promising complement to insecticide‐based malaria control interventions due to their effectiveness and safety (Fillinger & Lindsay, 2006; Fillinger, Ndenga, Githeko, & Lindsay, 2009). Coupled with the advent of long lasting formulated products with potential for sustained release of active ingredients, their use in integrated vector management (IVM) is likely to expand in the near future. However, application of formulations that last longer in the environment raises concerns with respect to their impact on non‐target organisms (Boisvert & Boisvert, 1999; Dupont & Boisvert, 1986). Of particular importance in mosquito larvae ecology is the safety of microbial larvicides on beneficial organisms that help in regulating mosquito density in aquatic habitats (Åbjörnsson et al., 2002; Bond et al., 2005; Kiflawi et al., 2003; Lima, 1998; Petranka & Fakhoury, 1991; Spencer et al., 2002). The current study monitored the impact of two LLML formulations (FourStar^®^ and LL3) comprised of a combination of Bti and Bs on non‐target organisms cohabiting with mosquito larvae when applied at a recommended dosage under operational malaria vector control in western Kenya highlands. It was expected that if LLML had any effect on non‐target organisms the outcome should be a decline in survival of non‐target organisms in the treated habitats.

In the current study, the abundance of eleven taxa of non‐target organisms studied was not significantly altered by application of either FourStar^®^ or LL3 LLML. The results thus corroborated with previous findings indicating a high level of safety of Bti and Bs to non‐target organisms cohabiting with mosquito larvae when applied at recommended rates (Brown, Watson, Carter, Purdie, & Kay, 2004; Lacey & Merritt, 2003; Lagadic, Roucaute, & Caquet, 2014; Merritt et al., 2005). Of particular relevance, no study has so far reported any direct significant effect of Bti and Bs to the organisms monitored in the current study. Significant adverse effects have been observed in certain dipterans when exposed to Bti, as summarized in a review by Boisvert (Boisvert & Boisvert, 2000). However, in most of these cases, treatments were either overdosed or the adverse effects were linked to other factors such as formulation additives, turbidity or methodological errors (Boisvert & Boisvert, 2000).

The present findings moreover revealed that application of FourStar^®^ or LL3 LLML did not alter richness or community diversity of the eleven taxa studied, when comparing the intervention and control larval habitats. Analysis by individual order of organisms did not indicate any significant alterations of population structure in treated and control larval habitats. However, application of FourStar^®^ or LL3 LLML caused a significant decline in malaria vectors (*Anopheles gambiae* complex and *An. funestus* group). This novel selective toxicity is based on the presence in Bti/Bs toxins, a receptor binding region believed to determine insect specificity (Lacey, 2007). The inherently high level of safety to non‐target organisms makes microbial larvicides not harmful to the environment and ideal for use in IVM operations (Walker & Lynch, 2007). Our findings thus agree with those of previous studies indicating a high level of effectiveness and safety of Bti and Bs when used for mosquito control (Fillinger & Lindsay, 2006; Fillinger et al., 2003; Fillinger, Ndenga, et al., 2009). Comparison of the activity of the two LLML formulations (FourStar^®^ and LL3) indicated that their efficacy against malaria mosquito vectors and their safety to non‐target organisms were not significantly different. Our findings thus suggest that FourStar^®^ and LL3 LLML have the potential for inclusion in the IVM even in areas with high levels of pyrethroid insecticide resistance like those of western Kenya where the current study was conducted (Wanjala et al., 2015). However, removal of target organisms (mosquito larvae) by Bti and Bs intervention may in the long run reduce the ecosystem diversity and alter the population structure of aquatic organisms cohabiting with mosquito larvae (Hershey et al., 1998; Merritt et al., 1991). This should call for regular monitoring of the long‐term direct and indirect impact of their application in the control of mosquitoes.

It has previously been reported that lentic organisms are particularly more exposed to Bti and Bs than lotic organisms due to heavier accumulation of toxin in the former than the later ecosystem (Boisvert & Boisvert, 2000). With mosquito larval habitats being of the lentic ecosystem, there may be a possibility of accumulation of Bti and Bs toxins to levels that can impact non‐target organisms. In this respect, analysis of the diversity of non‐target organisms per habitat type in treated and control larval habitats revealed two important scenarios. In relatively permanent larval habitats like abandoned gold mines, drainage ditches, ponds and fish ponds, the diversity of the studied organisms as expressed by Simpson diversity index values was stable throughout the monitoring period. However, in the temporary larval habitats (swamps and roadside canals), the diversity values for the organisms varied both in intervention and control arms. A possible explanation for this could be continuous changes in the dynamics of these temporary larval habitats such as drying and recurring after rains. It was evident that with low numbers of organisms in these particular habitats and low numbers of replications (contributed only 4.5% of total larval habitats surveyed), increase in abundance of one order of the organisms will result in increase in variation of diversity of the organisms. Despite of this variation in population diversity in temporary larval habitats, which was not related to the intervention (as it occurred in both treated and control sites), the general trend showed a lack of impact of FourStar^®^ or LL3 LLML to the non‐target organisms co‐occurring with mosquito larvae.

It is undisputable that chemical insecticides will remain an important malaria mosquito control intervention in a foreseeable future. However, their perceived risk to the environment, the emergence, and spreading of insecticide resistance and the possible change in mosquito dynamics has raised considerable attention to the search for alternative control agents (Federici, 1995). Thus, insecticide resistance and behavioral adaptations of malaria vector call for novel control methods that prevent or delay evolution of these traits. In this respect, the importance of microbial larvicides and their potential for inclusion to IVM strategies cannot be overemphasized. With their high level of safety to the environment, they preserve organisms that not only provide ecosystem services (food web & pollination) but also regulate mosquito proliferation through predation and competition.

## 5 CONCLUSIONS

Our findings suggest that one round of label rate application of LLML FourStar^®^ or LL3 in natural mosquito larval habitats will not alter the abundance or diversity of aquatic vertebrates and invertebrates cohabiting with mosquito larvae to an ecologically significant level. Our findings thus corroborate with previous reports indicating a high level of safety of products based on Bti and Bs and a potential role of their inclusion in integrated mosquito vector control programs. As these products are prone to accumulate in mosquito larval habitats and thus reduce the abundance of target organisms in the ecosystem, monitoring the long‐term impact of LLML products to the population structure of non‐target organisms is a crucial task for programs deploying them for mosquito control.

## CONFLICT OF INTERESTS

The authors declare that they have no competing interests.

## AUTHORS' CONTRIBUTIONS

YAD, SCK, FWM, EJK, HEA, XW, GZ, ML, AKG, and GY conceived and designed the study. YAD and SCK conducted field and laboratory experiments. ML managed spatial data, GZ and YAD performed data analysis. YAD drafted the manuscript with contributions from SCK, FWM, EJK, HEA, XW, GZ, ML, AKG, and GY. All authors read and approved the final version of the manuscript.

## DATA ACCESSIBILITY

All relevant data supporting the conclusion of this article are included within the article.
